# Struggling with capital: Recovery after severe traumatic brain injury among working‐age individuals in Denmark

**DOI:** 10.1111/hex.13946

**Published:** 2023-12-21

**Authors:** Anette L. Hindhede

**Affiliations:** ^1^ Department of Public Health University of Copenhagen Copenhagen Denmark; ^2^ UCSF Center for Health Research Copenhagen University Hospital Copenhagen Denmark

**Keywords:** capital, inequality, recovery, rehabilitation, transition, traumatic brain injury

## Abstract

**Objective:**

This study uses capital theory to investigate survivors' investments in their bodies and the resources they accumulate during their rehabilitation trajectory, and how these factors impact their perception of recovery from their impairments.

**Methods:**

Qualitative interviews were conducted with 20 patients of working age and their relatives, with audio recordings transcribed verbatim. Data analysis utilized an abductive approach informed by Bourdieu's capital theory.

**Findings:**

During the initial phases of rehabilitation (acute and subacute), survivors invest in their physical bodies and acquire physical capital. However, they encounter a range of complex barriers when attempting to convert this capital into the resources necessary for re‐entering the workforce or pursuing education. These difficulties are linked to the lack of specialized community services in the later phases of their rehabilitation trajectory.

**Discussion and Conclusion:**

Present‐day Danish healthcare rehabilitation focuses on restoring physical function and recovering physical capital. However, comprehensive rehabilitation to enhance mental and cognitive abilities and increase levels of emotional capital, which is crucial for working‐age individuals, is inadequately addressed within community services. This results in unequal treatment and care, contradicting the stated goal of equality in the Danish healthcare system.

**Patient or Public Contribution:**

This study incorporated the perspectives of 20 individuals who have survived severe traumatic brain injury, as well as their relatives, to gain insights into their experiences throughout the rehabilitation process, the resources they have accumulated and how these factors contribute to their sense of recovery.

## 1 INTRODUCTION

Traumatic brain injury (TBI) results from an external force impacting the brain. Survivors of TBI often face physical, mental and cognitive disabilities.^1^ Apart from relearning basic motor skills, individuals with TBI encounter challenges in short‐term memory, experience poor concentration and attention spans, undergo frequent mood fluctuations and struggle with motivation.^2^, ^3^ Rehabilitation serves as a fundamental strategy for managing TBI by recognizing the organism's potential for tissue damage recovery. Its objective is to help individuals regain their previous level of functioning to the greatest extent possible.

The Nordic countries boast extensive redistributive income policies and comprehensive universal state‐funded social and healthcare systems. Nevertheless, documented inequalities in illness, service provision and outcomes persist within these countries.^4^, ^5^ In Denmark, the incidence of severe TBI stands at approximately 2.3 cases per 100,000 individuals, predominantly affecting younger people of male gender.^6^ Consequently, individuals in the prime of their lives, coinciding with significant milestones such as completing education, developing careers and starting families, may face a profound deviation from their anticipated life trajectory.

Generally, TBI survivors and their relatives navigate a rehabilitation system that prioritizes younger adults.^7^ This is evident in the higher admission rates of younger patients to highly specialized rehabilitation facilities^8^ and the more frequent direct transfers from acute care to specialized rehabilitation.^9^

Despite this emphasis, TBI survivors still struggle with the consequences of their injuries, particularly in managing cognitive impairment.^10^, ^11^ Research indicates that the presence of family caregivers can help them to overcome barriers to accessing rehabilitation services.^12^, ^13^ However, studies have also revealed a significant prevalence of unmet needs among individuals with acquired brain injury, their caregivers and family members.^14^

We hypothesized that there may exist a discrepancy between the medical determination of recovery and what working‐age survivors and their relatives perceive as recovery.^15^ Thus, this study aims to answer the following research question: What types of resources do Danish working‐age severe TBI survivors accumulate during rehabilitation? And how do these resources relate to their own sense of recovery?

## 2 CONTEXT

According to the Danish Health Authority,^16^ TBI rehabilitation and recovery consist of four stages, including two hospital phases (acute and subacute) and two municipal phases (rehabilitation and stabilizing; see Table 1).

**Table 1:** Phases of rehabilitation after traumatic brain injury.^16^

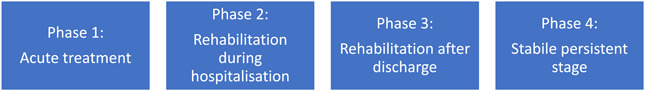

In the acute stage (see Table 1), the focus is on limiting the medical secondary consequences of brain injury (bleeding within the brain, build‐up of cerebrospinal fluid within the brain, infections if there is a skull fracture, etc.).^17^ Additionally, early rehabilitation and possible preventive treatment can be initiated. Based on an estimation of the severity of the injury and the needs of the patient, the patient then may be transferred between different departments (cardiology, orthopaedics and traumatology, infectious disease and oncology) as rehabilitation proceeds (Phase II). After discharge from the hospital, municipal rehabilitation takes over (in 2007, a municipal reform came into effect, transferring the responsibility for rehabilitation and recovery after hospital discharge to the municipalities) (Phase III). Again, the patient is assessed as requiring basic, advanced or specialized levels of rehabilitation based on the complexity and severity of the injury.

Following rehabilitation, as patients transition into a more extended phase (Phase IV), according to the Danish Health Authority, they can pursue various types of training, funded by the state, aimed at enhancing functions, preserving existing abilities or delaying functional decline.

Comprehending the distinct focus on physical, behavioural and cognitive issues within each of these four phases is essential for gaining a deeper insight into the resources accumulated during rehabilitation and the recovery process.

## 3 CAPITAL THEORY

A productive approach for understanding the accumulation of resources among TBI survivors is through the lens of Bourdieu's capital theory. Bourdieu posits that capitals are the outcomes of investments and resources that potentially can be utilized and transformed into other forms of capital.^18^ He uses the term ‘field’ to refer to a social arena or space where struggles for resources occur. In the case of working‐age TBI survivors, investing in their bodies becomes crucial for optimizing rehabilitation outcomes and achieving recovery. While previous studies have emphasized the significance of *social capital* in terms of family engagement,^19^, ^20^ there is limited knowledge about the specific types of investments necessary to recover from the physical, behavioural and cognitive impairments following a severe TBI, as these aspects significantly influence most aspects of everyday life.^21^ Physical capital is developed to consider how the physical body possesses value in various settings.^22^ In terms of the TBI survivor's sense of recovery from physical impairment, they invest in their physical body while relearning basic motor skills, relying on their accumulated physical capital when striving to regain preinjury functioning.

However, they may also experience cognitive difficulties, which are often regarded as invisible. These difficulties encompass a range of issues such as brain injury fatigue, memory problems, reduced concentration, challenges in planning and structuring everyday life, lack of self‐awareness and emotional instability.^23^ According to Illouz,^24^ *emotional capital* refers to the ability to make emotional connections to others or to be in touch with one's own emotions while maintaining sufficient control of them to avoid appearing driven by them. Investing in emotional capital after injury may be crucial for working‐age TBI survivors who aim to return to their preinjury work life. Additionally, for young working‐age TBI survivors pursuing educational success, accumulating *cultural capital* becomes particularly relevant. This encompasses both material and nonmaterial resources. Embodied cultural capital pertains to internalized dispositions acquired through socialization, while institutionalized cultural capital includes educational credentials. TBI survivors' educational aspirations involve both forms of cultural capital.

## 4 METHODS

This study is part of a larger project that involved analyzing data from the Danish Head Trauma Database (DHD) focusing on working‐age individuals (aged 18–60) who had experienced a TBI. TBI severity is categorized as mild, moderate or severe based on the duration of the loss of consciousness and posttraumatic amnesia.^25^ The DHD includes patients with *the most severe head traumas*—approximately 100 per year—who are admitted to one of the two national hospitals providing highly specialized rehabilitation after severe TBI.

To ensure that participants had undergone all four phases of rehabilitation, this study collected data from patients in the DHD database who had sustained their injuries 5 years prior. Furthermore, the inclusion criteria stipulated that, despite their severe traumatic injuries, participants must be able to engage in both a survey and an interview. To assess if survivors were able to decide participation, we used Rancho Los Amigos scale (RLA).^26^ RLA is a scale assessing level of cognitive functioning and is included in the DHD when the survivors are visiting the departments at 12 months follow‐up after TBI.

Out of 200 patients, 53 participated in the survey. Among them, 20 agreed to participate in the following in‐depth qualitative interviews. Thus, the following data were collected:

*Surveys* (*n* = 53) with questions on sociodemographics and social relationships before and after the TBI aimed to explore survivors' experiences 5 years postinjury with the rehabilitation system across all four phases and inquire about their bodily investments during rehabilitation. The survey also encompassed questions regarding the survivor's preinjury social network and its functionality in relation to the rehabilitation process and daily life postinjury. The analysis of this data is reported elsewhere.^6^ The average age of respondents was 35 years, and 70% identified as male. Respondents were geographically dispersed across Denmark, possessed diverse educational backgrounds and exhibited varying types of previous employment (see Table 2).

**Table 2:** Overview of participants.

Participant	Relatives present at interview	Age at the time of interview	Gender	Preinjury status	Status 5 years postinjury
R1	Wife	42	Male	Engineer and leader	Engineer and leader
R2	Wife	57	Male	Sales representative	Pensioner on disability
R3	Mother	27	Female	Sabbatical leave after high school	Just accomplished technical college degree
R4	Father	26	Male	Bachelor's degree student at university	Accomplished master's degree at university
R5	–	27	Male	Carpenter	Student at bachelor's degree level, university
R6	Parents	43	Female	University lecturer	Pensioner on disability
R7	Parents	22	Male	‘Troublemaker’	In work ability testing
R8	–	42	Female	Sales assistant	In work ability testing
R9	–	41	Male	Scaffolder	Disability pensioner
R10	–	28	Male	Construction builder	In work ability testing
R11	–	32	Male	Scaffolder	In work ability testing
R12	Son	50	Female	Lecturer	Disability pensioner
R13	Wife and son	45	Male	Director	Disability pensioner
R14	–	23	Male	Basic course at social and health school	Work ability testing at factory
R15	–	27	Male	Bricklayer apprentice	Bricklayer
R16	Father	43	Female	Office assistant	Pensioner on disability
R17	–	31	Male	Blacksmith	Flex job at a nursing home as caretaker assistant
R18	–	28	Male	Farmer	Pensioner on disability
R19	Husband	49	Female	Nurse	Pensioner on disability
R20	–	23	Male	High school student	In work ability testing

*Interviews* (*n* = 20) with participants scattered across Denmark were conducted by the author, an experienced researcher in qualitative methodology and public health. The interviews took place either with the TBI survivor alone or with a chosen relative, usually in their homes. The interviews focused on constructed narratives encompassing supportive elements and tensions related to the accident, the rehabilitation trajectory and the extent to which services met the respondent's needs and expectations regarding their sense of recovery. Over time, a complex pattern of partially differing perspectives emerged among the interviewees.

For the analysis, we employed an abductive approach to maximize the potential for surprising findings, aiming to defamiliarize the familiar to evoke abductive insights.^27^ To ensure the trustworthiness of the findings, I followed the approach specific to abductive research methodologies for conducting thematic analysis.^28^ First, I immersed myself in the data, reading and rereading the patient stories to gain a deep understanding of the material. I then conducted two rounds of coding, followed by the identification of themes and patterns in the patient stories. These themes and patterns were then compared in the context of existing theories. I integrated the identified themes and patterns with Bourdieu's capital theory to provide a comprehensive understanding of the observed phenomena.

An unanticipated empirical observation emerged, indicating that although the focus was on restoring survivors' physical capital, their cognitive functioning was left unaddressed in the rehabilitation trajectories. Bourdieu's forms of capital could not fully account for or explain these empirical findings. This prompted a small theoretical development as I turned to Illouz's theory of emotional capital to conceptualize this observation, providing a framework for further analysis of the forms of bodily investments during the hospital phase and after discharge.

I did not engage in member checking, a process where researchers discuss their findings with participants to ensure the accuracy and relevance of the analysis. However, I participated in peer debriefing sessions to discuss my analysis, receive feedback and refine my findings.

The study was conducted in accordance with the guidelines outlined in the Helsinki Declaration,^29^ and several ethical issues were taken into account. First, survivors and their families are vulnerable for several years following a TBI. Second, the research topic itself may evoke emotional distress. Given these factors, the author was particularly careful when selecting the inclusion criteria. Informed consent was obtained from both the survivors and their relatives before the interviews, and the participants were assured that their anonymity would be protected. Therefore, all names have been changed. The participants were also informed that they had the right to withdraw from the study at any time without any impact on their future treatment and care. Permission to access data from the DHD and to contact participants with brain injury was granted by the Danish Health Authority (https://www.sst.dk/en), and the necessary approvals were obtained. The project was also approved by the Danish Data Protection Agency (journal number 2015‐57‐001).

## 5 FINDINGS

All respondents had been diagnosed with severe brain injuries, and they had all spent several months at one of the two neurointensive departments in Denmark. In general, the respondents and their relatives explained how the injury in various ways was coinciding with important events such as developing careers or returning to work. During Phase I (see Table 1), relatives had numerous questions about the long‐term effects of the brain injury on the injured person's future functioning. They learned from hospital staff that the most rapid improvement occurs within the first 12 months after injury. Within that period, all progression needed to be made. One parent shared how they developed a close friendship with the hospital staff and learned ‘what to do so as not to have my son end up completely retarded’ (father to R‐4).

A shared experience among the survivors was that, during Phases I and II, hospital rehabilitation primarily aimed at helping patients regain lost physical capital through intensive physical training. However, as Phases III and IV approached, the responsibility for rehabilitation shifted to different municipal departments.^30^ In these later phases, both survivors and their family members sought additional improvements in the survivor's mental and cognitive capacities and increase levels of emotional capital, which are vital for pursuing education or re‐entering the workforce and the accumulation of cultural capital. During this period, they encountered less specialized rehabilitation programs and encountered perspectives on recovery that differed from their own.

### 5.1 Phases I and II: Investing in the physical body and accumulating physical capital

Throughout the entirety of Phase I and a significant portion of Phase II, when survivors were unconscious, they depended on support from their relatives. Family members would often quit their jobs to provide full‐time care for the injured individual, closely follow their treatment and maintain communication with health professionals. Although none of the relatives were trained healthcare professionals, they learned by listening intensely to the advice and actions of healthcare professionals. One parent shared their experience about their daughter:

Her body had to rediscover what it could really do—everything, from walking to using her hands. When you hit your head, you lose the ability to produce the hormons that keep us going. So, we pushed her into training. My child has damaged her frontal lobe—she's lost it. So, feelings and all that; she does not have much of that. (Mother to R‐3)

They also learned how there were significant differences between the rehabilitation services offered in different municipalities. The parent described their encounter:

The chief neurologist was a very pleasant man, really worried about my daughter, and pressured the highly specialised rehabilitation centre to find room for her (…) they always sought the best for my daughter because she was so young, and needed to be rehabilitated as quickly and as well as possible because it was so important for her to come back… I cannot say to a normal life because it is not, but to a life that is tolerable for my daughter. (Mother to R‐3)

Another common experience shared by survivors and their relatives was that during phases I and II, hospital staff refrained from making any definitive assurances about the future. One survivor articulated this frustration by saying:

When I received this diagnosis, nobody provided any concrete information. Not even a highly trained neurologist. They simply stated, ‘The status is… It can go in this direction or that direction’. That's about as much as you could extract from them. I was almost begging professionals for something tangible to grasp. I had no understanding of what was unfolding, and no matter whom I turned to—my mother, my girlfriend, my father, my closest friends—nobody had any answers. (R‐5)

One of the respondents, R‐4, visualized the process in Phases I and II as a factory where a patient entered at one end and then emerged from the other with the result: ‘It all depends on the kind of resources and the network you have and how you can put it into play’. R‐4 commented that if he had only one parent, or a single parent with his two siblings also in need of care, his family would not have had the capacity to closely follow his rehabilitation and encourage him to prevent him from becoming like others at the ward: ‘Otherwise, I would not be sitting here this way today. I probably would not have been sitting here at all. So it is alpha and omega, the resources you have around you’ (R‐4).

### 5.2 Phases III and IV: Moving from one understanding of recovery to another

Despite the significant amount of time relatives spent with the survivor during the hospitalization phases, the demands placed on them in Phase III, after the survivor's discharge, became even more taxing. During this phase, the cognitive deficits became more apparent. As one relative expressed about his son's unresolved impairment in mental processes which prevented him from independently acquiring information and knowledge, instead having to rely on relatives to facilitate cognitive rehabilitation:

When you are discharged, you move to the phase where rehabilitation must be done as cheaply as possible (…) and I simply did not accept what our municipality offered (…). Many would break down mentally because you cannot win this fight if you are not endlessly resourceful’. (R‐5)

As relatives were informed by healthcare staff in the highly specialized rehabilitation departments that they had only 12 months to act, they persistently addressed their municipality's insufficient rehabilitation services following the patient's discharge. While municipalities typically provided survivors with the opportunity to participate in community‐based group exercise programs to enhance their physical well‐being, it was evident to all relatives that much more needed to be accomplished.

One illustrative instance of these challenges involves a father who described how, after discharge, his son would passively sit in a chair and respond with a simple ‘yes’ to everything. The father had been advised by hospital staff that his son required intensive training from a neuropsychologist, but the municipality did not offer this type of rehabilitation. The father then argued that the municipality should cover the expenses for specialized neuropsychological rehabilitation in another municipality. However, the municipality declined this request. One day, the son received an invitation from a specialized care centre for a stay. Due to budget cuts in many municipalities, patient referrals to this institution decreased, affecting its revenue. To prevent closure, the centre started offering free stays to individuals, irrespective of their municipality.

Throughout the cases, survivors and their family members encountered challenges in pursuing their future plans. These challenges arose from discrepancies between existing rules and regulations and the differing understandings of recovery held by community workers. For instance, in Phase III, survivors were required to collaborate with a brain injury coordinator to enrol in further education or secure employment. However, these coordinators often hindered the process by judging that the survivors were not fit enough for school or employment.

Initially, TBI survivors were obligated to demonstrate their ability to work 20 h a week. As a result, they were sent for various work trials every 3 months for an extended period, despite facing difficulties in adapting to new environments due to their cognitive impairment. The respondents and their relatives encountered a reluctance on the part of social workers to customize the workplace for the individuals with brain injuries to address the challenges they faced. Consequently, issues linked to the survivors' cognitive functioning were left unaddressed. For instance, there was no assessment of tasks to identify opportunities to exclude complex activities or break them down into smaller components. Additionally, there was no review from the municipality regarding reduced work tasks, a more defined structure in the workday, fewer daily working hours or longer breaks during the workday. A relative shared their experience when their son was nearing discharge from the hospital. Health professionals advised them to hire an injury lawyer. Following this advice, the lawyer warned him, ‘You must be aware that a dark horse may come here in the end’ (father to R‐4). And indeed, the Labour Market Insurance was responsible for evaluating their son's accident, a process that spanned nearly 2 years, encompassing both Phases III and IV. The relative expressed:

I believe it's simply a matter of wearing people out because they have numerous avenues to pursue. When you require compensation for necessary home renovations or assistance that you cannot afford, you feel powerless as they employ various tactics to delay progress. (Father to R‐4)

## 6 ACCUMULATING EMOTIONAL CAPITAL AFTER DISCHARGE

The behavioural changes resulting from TBI initially caused all TBI survivors to experience various forms of alienation from their family and friends. Moreover, in cases where relatives were present during the interviews, they frequently described how the TBI survivor could transform into a ‘challenging stranger’ on occasion. For all respondents, TBI had altered their sense of self. Some respondents experienced compromised abilities to accurately interpret social cues and solve social problems. They shared instances where their close relationships were affected by unexpected aggressive behaviour and lack of empathy. Misconceptions about the effects of TBI among families, coworkers, and healthcare professionals sometimes exacerbated the situation. However, municipalities did not specifically address these behaviour changes for remediation. To foster independence and mitigate his son's maladaptive social behaviour (irritability and combative outbursts), one father explained that he personally financed his son's private behavioural management assistance. Another respondent acknowledged that he used to have good relationships with people but struggled to maintain them since the accident. He was aware of his lack of a social filter and described his interactions with others:

Everything is going completely wrong, and I struggle to get along with my friends. I end up saying foolish things and crossing boundaries without realising it. I don't know when to stop—I just keep going. Initially, people get annoyed, and before I can understand my mistake, they become furious with me. (R‐5)

## 7 CONVERTING ACCUMULATED CAPITAL TO CULTURAL CAPITAL

In addition to the increased levels of physical capital gained through intensive physical training during hospitalization, young adult survivors faced persistent cognitive impairments that posed significant challenges when attempting to return to work or continue their studies. Both survivors and their relatives highlighted how these cognitive impairments hindered the survivor's ability to observe and reflect on their own thoughts and actions. This was distressing for survivors as they struggled to comprehend why they were being restricted from certain activities.

For instance, during interactions with municipal social workers, TBI survivors often exhibited an ‘overly optimistic sense of self‐confidence’ that was deemed ‘highly unrealistic’ (R‐17). This was evident in their expectations of returning to their preinjury jobs. Consequently, social workers had very low expectations regarding the survivors' capabilities regardless of job type and demands. The same was experienced as for the level of education level before the TBI. One family member explained, ‘They didn't expect someone with my daughter's diagnosis to successfully complete higher education’ (mother to R‐3). Another respondent shared his experience after being unable to return to his previous job as a construction site leader and expressing a desire to pursue further education:

I wasn't allowed to pursue any form of study. They claimed that I hadn't proven my aptitude for learning [laughing]. I decided to start with a web‐based e‐learning course, but it turned out to be an incredibly arduous process. Tasks that a regular student would complete in five hours per week took me 37 hours. (R‐5)

Despite facing challenges in convincing his social worker of his suitability for learning, R‐19 received support from an individual at the local rehabilitation centre. With their assistance, he managed to persuade the municipality to allow him to enrol in one high school class at a time, aiming to obtain a complete high school diploma. However, this proved to be highly demanding for him as he did not find the study organized with built‐in breaks and support for homework and assignments. Additionally, there was no assistance in achieving a balance between student life and youth life. Eventually, he informed the centre's staff that he could not handle it any longer. He decided not to continue confronting the municipality, stating:

I reached out to my lawyer and informed them that I couldn't obtain any further assistance from the municipality. So now we'll pursue early retirement. If they refuse to help me, that's the path we must take. (R‐5)

The assistance provided by the municipality conflicted with his individual perception of recovery. Most importantly, he placed a higher value on engaging in meaningful activities rather than staying at home. His perspective shifted after he participated in a philosophy course where he was introduced to the work of the cognitive psychologist Howard Gardner. This experience allowed him to gain a deeper understanding of himself, and he expressed:

The (rehabilitation) system is incomprehensible for ordinary people. It's absurd. What my neuropsychologist did not want to share with me, I found in a [Gardner] book. Philosophy provided insights about life and society that no one else could offer me. (R‐5)

An example of a respondent with a different experience is R‐3, who strongly dislikes it when people assure her that her situation will improve. Health professionals had informed her that after a year, the brain's capacity for further rebuilding diminishes. R‐3 expressed her frustration, stating, ‘So, when people say to me, it'll probably get better, seriously, I feel like slapping them—because no, I know it won't’ (R‐3).

She further elaborates on the challenges she faced when justifying her desire for an education, recounting a conversation with the social worker (where the social worker had asked her why she was so keen on be able to work 20 h/week to be allowed to pursue education):

I looked at her one day and said: ‘Well, why did *you* pursue an education? Why do *you* want a job? My life is 180 degrees different from what I had hoped for—do you think I should just give up and rely on financial support from you? No thanks’. (R‐3)

She aspired to be like everyone else, to excel in something and earn a living on her own terms. However, she eventually realized that it was much easier to simply request early retirement.

In summary, the link between each strategy and the corresponding form of capital is emphasized in Table 3 below to illustrate the interplay between these elements in the rehabilitation process.

**Table 3:** Overview of strategies employed by TBI patients and their relatives during rehabilitation.

Strategy	Forms of capital involved	Strategy related to capital investment
Investing in the physical body during hospitalization (Phases I and II)	Physical capital	Patients and relatives invest in physical capital by actively participating in physical training and rehabilitation during the acute and subacute phases of TBI rehabilitation. Physical capital is crucial for regaining lost physical abilities.
Overcoming challenges in transition to municipal rehabilitation (Phases III and IV)	Cultural capital	Patients and relatives encounter challenges in accessing education and employment opportunities after hospital discharge (Phase III). These challenges relate to struggles with municipalities when seeking to convert gained physical capital into cultural capital. Social workers may have low expectations regarding the severe TBI survivor's capabilities, which affects survivors' ability to pursue higher education or return to work. Phase IV is a nonexistent ideal of the rehabilitation trajectory.
Accumulating emotional capital	Emotional capital	Emotional capital involves the ability to make emotional connections and manage emotions. Survivors encounter emotional challenges, including behavioural changes and altered self‐identity after TBI. Survivors and relatives seek to regain emotional capital to navigate changes in relationships, cope with behavioural issues and foster independence in work and education postinjury. However, cognitive rehabilitation is not included in the rehabilitation offered by municipalities, so survivors must pay themselves to get this.
Navigating the healthcare system	Social capital	Support from relatives is a critical form of social capital. Relatives provide care, advocacy and emotional support, assisting survivors in their rehabilitation trajectory. They actively participate in the patient's treatment and recovery process.
Dealing with municipality and social workers	Cultural capital, social capital	Survivors and relatives engage with municipal services and social workers to access posdischarge resources. They must navigate complex systems and negotiate with social workers who may have different expectations about the survivor's abilities. Cultural capital, such as educational aspirations, influences the survivor's path to education and work, and social capital is essential in these negotiations.

Abbreviation: TBI, traumatic brain injury.

## 8 DISCUSSION

For all respondents, the transition between rehabilitation phases entailed a decrease in both intensity and specialized expertize. Rehabilitation services in Phases I and II primarily concentrated on addressing their functional and physical impairments, thus accumulating physical capital. In Phases III and IV, there was significant variation in how local municipalities addressed the challenge of providing rehabilitation to patients. Primarily, they referred to non‐TBI‐specific rehabilitation, such as physiotherapy. These findings align with concerns raised more than 10 years ago by a group of specialists in Danish neurorehabilitation departments when the municipal reform from 2007 came into effect, transferring the responsibility for rehabilitation and recovery after hospital discharge to the municipalities. They experienced that half of the patients faced challenges with municipal services, particularly young adults sent for job trials without any rehabilitation support.^31^ According to the specialists, this misunderstanding arises as municipal caseworkers mistakenly perceive patients as having mental health issues, attributing their reduced motivation to work to fatigue and concentration difficulties. According to the specialists, well‐functioning young individuals, therefore, often risk ending up on social welfare due to either inadequate rehabilitation offers or none at all.^32^ Thus, according to the specialists, the Danish Health Authority allows for the ‘atomization’ of the neuroprofessional level by delegating the neuroprofessional responsibility even to the smallest municipalities.^32^ In this study, the willingness of municipalities to fund holistic neuropsychological rehabilitation seems to be decreasing, leading to specialized centres being at risk of closure.^33^ This risk is illustrated by a respondent who, by chance, was offered a free stay in one such centre.

As for the participants in this study, all diagnosed with severe TBI the level of education and employment status before the TBI had minimal impact on postinjury work/study abilities. The reason for this is that social workers did not consider the cognitive challenges when referring survivors for job testing (as they all had physical capabilities). Research indicates that even in cases of minor brain injury, similar outcomes are observed. In relation to returning to work, workplace accommodations, including reduced working hours, modified working conditions and support from coworkers, are found to be lacking.^34^ Furthermore, municipal case management is inadequate, as social workers lack sufficient knowledge regarding persistent post‐TBI symptoms.^34^

A recent Danish national development project within brain injury rehabilitation, funded by earmarked funds from the Danish Health Authority and carried out from December 2017 to 2021, similarly indicates that patients and their relatives experienced the need to advocate for themselves to receive services in the municipalities.^35^

In this study, social workers faced uncertainty, for example, when dealing with young TBI survivors who expressed desire to continue their studies. For instance, the story of R‐5 implies that the system does not anticipate young individuals like him to hold aspirations for the future beyond receiving a pension. Thus, decisions regarding access to rehabilitation following TBI are the result of a complex process that involves the interpretation of both clinical and nonclinical factors, as well as negotiations with healthcare professionals,^36^ aligning the findings of Norman and colleagues.^14^

To increase levels of cultural capital, TBI survivors need an emotional style that enables them to establish social relationships (i.e., social capital) that can support them in achieving their goals. The TBI survivors in this study were confronted with a significant deviation from their anticipated life trajectory. In several ways, they struggled with incorporating their frustrations related to cognitive challenges into their sense of personal growth and achievement. The strategies employed were shaped by their specific forms and levels of capital, the demands of their situations, and the unique paths of their personal trajectories. Consistent with other studies, support from relatives was also found to be important. All younger adults had parents who supported them throughout the initial stages of rehabilitation by temporarily leaving their jobs. This led to a reconfiguration of family relationships, with parents assuming the role of primary caregivers for their adult children. They became knowledgeable about healthcare practices, weighed different advice on behalf of their children and managed the emotions of other family members. This process was emotionally draining for the parents in this study.

The Danish rehabilitation field encompasses the health, labour and welfare sectors, leading to variations in conceptions of health, work and rehabilitation across different municipalities and institutions. Additionally, municipalities vary in their prioritization of different aspects of rehabilitation, creating tensions with the ideals of equality. The families observed how understanding of brain injury influenced the actions of municipalities and their social workers during phase III of the rehabilitation trajectory, thereby shaping and reshaping the identities of TBI survivors and the conditions under which they could thrive. Despite achieving the physical functioning goals set by the Danish neurorehabilitation system, the TBI survivors in all cases perceived themselves as part of a marginalized group and experienced a sense of devalued status. They faced challenges in applying their acquired capitals within the rehabilitation context due to the system's specific requirements. Specifically, lower levels of emotional capital impeded their efforts in pursuing education or employment, and this obstacle could only be overcome by high levels of social capital, such as support from close relatives.

For this study, I conducted a survey along with 20 interviews. While such a small sample size has been criticized for its limited generalizability, as argued by Mjøset,^37^ ^, p.47^ the contextualist strategy of generalization entails generalizing within specified contexts. Thus, specification and generalizations are not contradictory.^37^ ^, p.52^ The generalizability of my findings lies in the proposed understandings as frames of interpretation. The analyses are generalizable to the extent that the identified understandings are shared among other practitioners and scholars, either in other parts of the world or in future scenarios.

## 9 CONCLUSION

This study reveals disparities in the recovery trajectories of working‐aged TBI survivors. Challenges arise from deficiencies in community rehabilitation, a lack of specialized knowledge regarding TBI survivors' ongoing cognitive rehabilitation needs and varying financial investments across municipalities, resulting in health inequalities. Examined through capital theory, the study underscores the difficulty TBI survivors and their relatives face in converting physical capital acquired during hospitalization to the postinjury context, requiring cultural and emotional capital. Although acute neurorehabilitation in Phases I and II excels, the transition to community rehabilitation in Phases III and IV detrimentally affects the journey of TBI survivors, resulting in a disparity in the understanding of recovery.

This study emphasizes the necessity for increased focus on emotional capital, acknowledged as a new form of cultural capital, particularly for working‐aged individuals with severe TBIs. Emotional capital proves vital in negotiations with municipal staff, underscoring the survivor's capacity for learning, among other aspects.

## AUTHOR CONTRIBUTION

**Anette L. Hindhede**: Conceptualization; methodology; validation; investigation; writing—original draft; writing—review and editing.

## CONFLICT OF INTEREST STATEMENT

The author declares no conflict of interest.

## ETHICS STATEMENT

Informed consent was obtained from both the survivors and their relatives before the interviews, and the participants were assured that their anonymity would be protected. The participants were also informed that they had the right to withdraw from the study at any time without any impact on their future treatment and care. Permission to access data from the Danish Head Trauma Database and to contact participants with brain injury was granted by the Danish Health Authority (https://www.sst.dk/en), and the necessary approvals were obtained. The project was also approved by the Danish Data Protection Agency (Journal No 2015‐57‐001).

## Data Availability

Research data are not shared.
